# *Fascin-1* Promoter Activity Is Regulated by CREB and the Aryl Hydrocarbon Receptor in Human Carcinoma Cells

**DOI:** 10.1371/journal.pone.0005130

**Published:** 2009-04-02

**Authors:** Yosuke Hashimoto, David W. Loftis, Josephine C. Adams

**Affiliations:** 1 Department of Cell Biology, Lerner Research Institute, Cleveland Clinic, Cleveland, Ohio, United States of America; 2 Department of Molecular Medicine, Cleveland Clinic Lerner College of Medicine, Cleveland Clinic, Cleveland, Ohio, United States of America; Texas Tech University Health Sciences Center, United States of America

## Abstract

**Background:**

Fascin is an actin-bundling protein that is absent from most normal epithelia yet is upregulated in multiple forms of human carcinoma, where its expression correlates clinically with a poor prognosis. How *fascin-1* transcription is activated in carcinoma cells is largely unknown, although the hypothesis of regulation by β-catenin signaling has received attention. The question is important because of the clinical significance of fascin expression in human carcinomas.

**Methodology/Principal Findings:**

Through comparative genomics we made an unbiased analysis of the DNA sequence of the *fascin-1* promoter region from six mammalian species. We identified two regions in which highly conserved motifs are concentrated. Luciferase promoter reporter assays for the human *fascin-1* promoter were carried out in fascin-positive and -negative human breast and colon carcinoma cells, and in human dermal fibroblasts that are constitutively fascin-positive. In all fascin-positive cells, the region −219/+114 that contains multiple highly conserved motifs had strong transcriptional activity. The region −2953/−1582 appeared to contain repressor activity. By examining the effects of single or multiple point mutations of conserved motifs within the −219/+114 region on transcriptional reporter activity, we identified for the first time that the conserved CREB and AhR binding motifs are major determinants of transcriptional activity in human colon carcinoma cells. Chromatin immunoprecipitations for CREB, AhR or β-catenin from extracts from fascin-positive or -negative human colon carcinoma cells identified that CREB and AhR specifically associate with the −219/+114 region of the *FSCN1* promoter in fascin-positive colon carcinoma cells. An association of β-catenin was not specific to fascin-positive cells.

**Conclusion:**

Upregulation of fascin-1 in aggressive human carcinomas appears to have a multi-factorial basis. The data identify novel roles for CREB and AhR as major, specific regulators of *FSCN-1* transcription in human carcinoma cells but do not support the hypothesis that β-catenin signaling has a central role.

## Introduction

Abnormalities of the actin cytoskeleton make important contributions to the ability of carcinoma cells to invade adjacent tissue and metastasise via the blood or lymphatic systems to remote body sites [1]. Many of the actin-associated proteins that are reported to be upregulated in carcinomas, for example ezrin, are also expressed in the corresponding normal epithelium [2], raising uncertainty over their practicality as possible therapeutic targets. In recent years, the actin-bundling protein fascin has emerged as a functionally relevant mediator of carcinoma cell migration, invasion and metastasis in cell culture and mouse models [3]–[8]. Fascin (also known as fascin-1; gene name *FSCN1* in human and *Fscn1* in mouse) bundles F-actin into tightly packed parallel arrays that contribute to cell migration by providing rigidity to filopodia and microspikes [9]. Fascin is of considerable interest as a biomarker or potential therapeutic target because it is not expressed by simple epithelia and is low or absent in stratified epithelia, yet is strongly upregulated in most forms of human carcinoma [10]–[15]. Notably, in all forms of human carcinoma examined to date, high tumour expression of fascin protein is of clinical significance and is associated with a poor prognosis in carcinomas of the lung, oesophagus, stomach, colon, breast and kidney [5], [11], [12], [14]–[17]. Fascin expression has also been correlated with local lymph node metastasis and distant metastasis [12], [16]–[18].

Increased levels of *fascin-1* transcript have been reported in multiple human carcinomas (e.g. [10], [13]). The mechanism by which *fascin-1* transcription is upregulated in carcinomas is not understood. Normal mammalian cells with high levels of *fascin-1* transcripts include dendritic cells and neuronal cells [19]–[21] and initial analyses of mechanisms for transcriptional regulation have been made in these cells. In human or mouse Langerhans and dendritic cells, fascin is absent from immature cells and becomes highly expressed during terminal differentiation; this process involves increased levels of the *fascin-1* transcript [22], [23]. As a structural component of dendrites, fascin contributes to the antigen-presenting activity of mature dendritic cells [22], [24]. A 2.6 kb 5′ flanking region of mouse *fascin-1* is sufficient to drive promoter reporter activity in mature mouse dendritic cells but not in immature cells; similarly, a 3.1 kb 5′ flanking region of human *fascin-1* specifically confers induction of reporters in mature human dendritic cells and other non-transformed fascin-positive cells. No transcriptional regulation activity was detected in the first intron or 3′ untranslated region [25], [26]. In NT2 human neuronal precursor cells, the levels of *FSCN1* transcript and protein increased during retinoic acid-induced terminal differentiation. This effect depended on the transcriptional coactivator and histone acetyltransferase CBP (cAMP response element binding protein (CREB)-binding protein). However, the relevance of CBP to *FSCN1* promoter activity was not addressed in this study [27].

Dysregulation of the Wnt/β-catenin signalling pathway is a frequent cause of tumour progression in colorectal carcinomas. The transcriptional activity of β-catenin is mediated through its interaction with members of the TCF/LEF (T cell factor/ lymphocyte enhancer-binding factor 1) family of DNA-binding transcription factors [28]. There are conflicting reports on whether the transcriptional activity of *fascin-1* is regulated by β-catenin signalling [3], [8]. Grothey et al. [3] identified multiple candidate T cell factor (TCF) binding sites in the (mouse) *Fscn1* promoter, but found no effect of either TCF or β-catenin over-expression on *Fscn1* promoter activity when tested in human MDA-MB-435 cells. In contrast, a later study demonstrated regulation of *Fscn1* promoter reporter activity upon expression of either a stabilised β-catenin or dominant-negative TCF4 in two human cell lines [8]. Because both of these studies focused on analysis of the mouse *fascin-1* promoter, it remains unclear whether *FSCN1* could be a direct target of β-catenin/TCF transcriptional regulation in human carcinomas. Understanding the mechanisms of *FSCN1* transcriptional regulation in human carcinoma cells is an important question because it may lead to novel prognostic tools for early identification of the most biologically aggressive carcinomas, and/or potential novel therapeutic strategies to reduce tumour metastasis through inhibition of fascin expression. Here, we first took the unbiased approach of identifying evolutionary conserved regions of the *fascin-1* promoter region in mammals by comparative genomics. We built on these data to analyse experimentally the *cis*-acting mechanisms of *FSCN1* transcriptional regulation in human colon and breast carcinoma cells. Our novel findings demonstrate previously unidentified roles of cAMP response element-binding protein (CREB) and the aryl hydrocarbon receptor (AhR) in the regulation of *fascin-1* transcription in human carcinoma cells.

## Results

### Phylogenetic analysis of conserved motifs in the 5′ flanking region of mammalian *fascin-1* genes

To identify evolutionarily conserved sequences in the 5′ flanking region of mammalian *fascin-1* genes, we extracted 5.5 kb of DNA sequence 5′ to the ATG codon of the *fascin-1* gene from the genomes of human and five other mammals: *Pan troglidytes*, *Macaca mulatta*, *Canis lupus familiaris*, *Mus musculus* and *Rattus norvegicus*. For the rat and chimpanzee, a full sequence for the entire 5.5 kb region is not yet available, thus our detailed analysis focused on a 2 kb 5′ flanking region for which all six species could be fully aligned. The chicken (as an example of an avian) could not be included because this region of its genome is not yet sequenced completely.

To examine sequence conservation across the 2 kb 5′ flanking regions, a TCOFFEE multiple sequence alignment was prepared (Figure S1). The alignment demonstrated that the three primate genome sequences are near identical throughout this region apart from scattered individual single nucleotide differences and some short additional sequences in the macaque. In contrast, the dog and rodent sequences contained multiple nucleotide insertions and /or deletions, resulting in multiple gap insertions throughout the alignment (Figure S1). Overall, the sequence conservation between the 5′ flanking regions of human and rodent *fascin-1* was much lower than between the primates (for example, the human and mouse sequences have 54.6% identity over the 2 kb region, whereas human and macaque are 90.5% identical). The most significant region of conservation across all six species was within a 200 bp region, 5′ to and including the translational start site (Figure S1).

**Figure 1:**
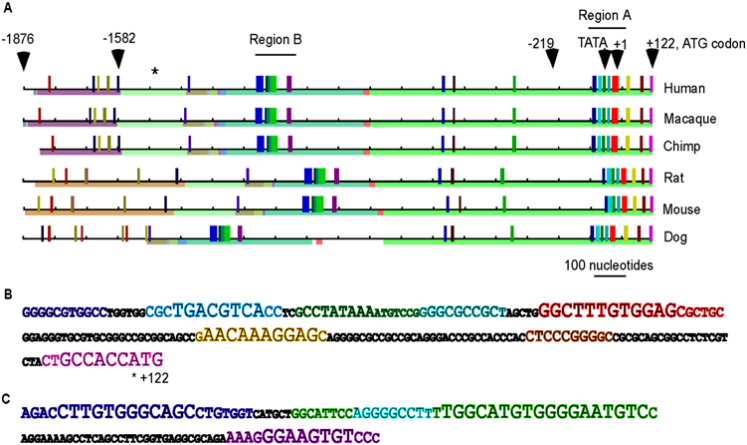
Identification of evolutionarily conserved motifs in the *fascin-1* promoter region by phylogenetic footprinting analysis. A, 2 kb of genomic DNA sequence 5′ to the translational start codon of *fascin-1* was extracted from the indicated mammalian genomes and analysed for conserved motifs by the FootPrinter algorithm. On each line, alignment blocks are indicated by thin coloured lines. The vertical bars indicate the positions of motifs that are conserved across the genomes, with each category of motif identified in a different colour. Corresponding motifs in different sequences are in the same colour. The numbering scheme is the same as that used in other figures, with the transcriptional start site in the human genome as +1. Asterisk indicates the position of a TCF motif identified by the rVista algorithm that is conserved in the human, chimpanzee and dog genomes. B, the DNA sequence and conserved motifs within Region A from the human genome. C, the DNA sequence and conserved motifs within Region B from the human genome. In B and C, motifs are in the same colour code as in A. The larger the font the stronger the conservation of the motif: the largest font indicates complete conservation in all six species.

For an independent second methodology, the six sequences were analysed by phylogenetic footprinting with the algorithm FootPrinter. This algorithm identifies the most conserved nucleotide motifs within a set of homologous DNA sequences, under the premise that functional regulatory elements are more highly conserved under natural selection than non-functional DNA sequences [29]. The “footprints” obtained demonstrated complete conservation of multiple motifs within the 200 nucleotide region 5′ to the translational start site (Fig. 1, designated region A). A second region that included a high density of well-conserved motifs was located between −1200 and −1000 in the three primate sequences (Fig. 1, designated region B). Within region B, the same motifs were conserved in all six species, but, in agreement with the gapping noted in the TCOFFEE alignment, the exact positioning of the motifs relative to the start site was different in the rodent and dog sequences compared to the primates. Further 5′ to region B (i.e., between −1876 to −1176), the three primate sequences contained additional conserved motifs in near-identical locations relative to the translational start site (Fig. 1A). The rodent and dog sequences had different numbers and locations of conserved motifs within this region (e.g., the absence of blue-coded motifs in region −1676/−1576, Fig. 1A).

### Mapping *FSCN1* transcriptional activity in fascin-positive and fascin-negative human carcinoma cells

**Figure 2:**
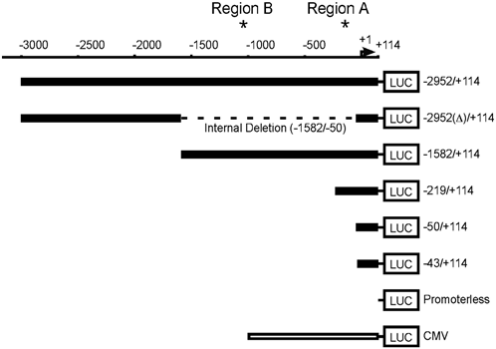
Schematic diagram of *FSCN1* promoter-luciferase reporter constructs. The scale bar indicates the *FSCN1* 5′ flanking region. The name of each construct corresponds to the 5′ and 3′ junctions within the *FSCN1* promoter, relative to the proposed transcriptional start site. The numbering scheme is the same as in Fig. 1. Broad black lines indicate *FSCN1* 5′ flanking regions; dashed line represents an internal deletion. Thin black lines represent sequence from pcDNA3.1, 5′ to the firefly luciferase cDNA.

On the basis of the comparative genomic analysis, we hypothesised that either or both of regions A and B might have significant regulatory roles in controlling *FSCN1* transcription in human carcinoma cells. To test this idea, we generated a set of luciferase reporter constructs to compare the activity of a 3.1 kb 5′ flanking region of *FSCN1* (comprising the region −2956/+114) with truncation mutants that included or deleted either or both of regions A and B, or separated these regions from the distal conserved motifs. A CMV promoter-driven luciferase reporter was included as a positive control and a promoter-less luciferase as a negative control (Fig. 2).

**Figure 3:**
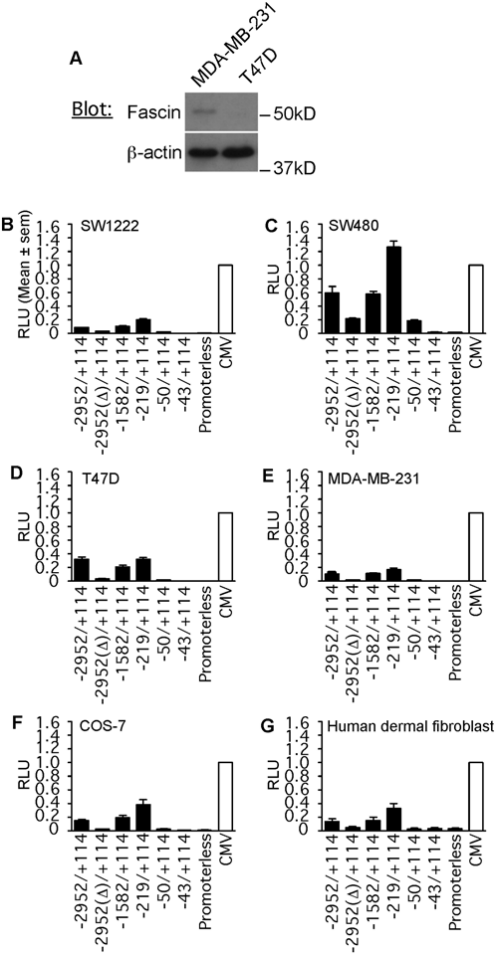
*FSCN1* promoter reporter activity in fascin-positive and -negative cells. A, comparison of fascin protein levels in MDA-MB-231 and T47D breast carcinoma cells. Whole cell extracts from the equivalent of 4.5×10^4^ cells/lane were resolved on 10% SDS-PAGE gels under reducing conditions, transferred to PVDF membrane and immunoblotted with the indicated antibodies. β-actin was used as a loading control. B–G, *FSCN1* and CMV promoter activities were analysed by dual luciferase reporter assays in the six indicated cells. In each experiment, firefly luciferase activities were normalized against Renilla luciferase activity. In each graph, the mean normalized CMV promoter activity for that cell type is set as 1 and FSCN1 reporter activities are expressed as a fraction of the CMV reporter activity. Each column represents the mean from 3 independent experiments, bars indicate s.e.m.

The luciferase reporter activity of these constructs was tested in cells derived from human colon and breast adenocarcinomas, because fascin expression is strongly associated with a poor prognosis in these carcinomas [14], [15], [17]. The human colon adenocarcinoma cell line SW480 has high fascin expression, whereas SW1222 has negligible expression [4], [7]. T47D is a well-differentiated, fascin-negative carcinoma cell line derived from an invasive ductal carcinoma [30] and MDA-MB-231 is an invasive breast carcinoma line [31] with moderate fascin expression (Fig. 3A). For comparison with promoter activity in a normal cell type, we examined human dermal fibroblasts (HDF) that are constitutively fascin-positive. COS-7 green monkey kidney cells were included for comparison as fascin-positive, [32], transformed primate cell line of epithelial origin.

In all cells, the CMV promoter was more active that the 3.1 kb *FSCN1* promoter. When normalized relative to CMV promoter activity, the highest reporter activity of the 3.1 kb *FSCN1* promoter was detected in SW480, that have the highest level of fascin protein (Fig. 3B, 3G). SW1222 cells or T47D do not express fascin protein, yet a low level of 3.1 kb FSCN1 promoter-luciferase reporter activity was detected in these cells (Fig. 3C, 3D). MDA-MB-231, COS-7 and HDF cells, with moderate expression of fascin, had similar intermediate levels of luciferase reporter activity (Fig. 3E–3G). The level of 3.1 kb *FSCN1* reporter activity in COS-7 cells appeared low as a ratio against CMV promoter activity because of the very high level of CMV promoter activity in these SV40-transformed cells (Fig. 3G).

With regard to the deletion mutants, in SW480 cells, the distal 5′ flanking region −2952(Δ)/+114 activated less than half the luciferase reporter activity of the 3.1 kb *FSCN1* promoter (Fig. 3B). A similar fold reduction in activity was observed in all other cell lines tested (Fig. 3B–3F). In contrast, the reporter activity of the −1582/+114 region was equivalent to that of the 3.1 kb *FSCN1* promoter in all the cells (Fig. 3). In all cells except T47D the −219/+114 promoter fragment, that includes only conserved region A, had significantly increased reporter activity compared to the 3.1 kb *FSCN1* promoter or the −1582/+114 region (Fig. 3; *p* = 0.0002 for SW480 cells). Further truncation to −50/+114, thus deleting region A, dramatically reduced luciferase reporter activity in SW480 and all the other cells (Fig. 3). Residual luciferase reporter activity in the carcinoma cells was completely abolished by deletion of an additional seven nucleotides: the construct −43/+114 had the same negligible activity as a promoterless luciferase reporter (Fig. 3). These results demonstrated that positive regulatory sequence elements active in human carcinoma and fibroblast cells are concentrated within the region −219 to −43 of *FSCN1*.

### Transcriptional activation by the −219/+114 region of *FSCN1* depends on multiple DNA sequence elements

**Figure 4:**
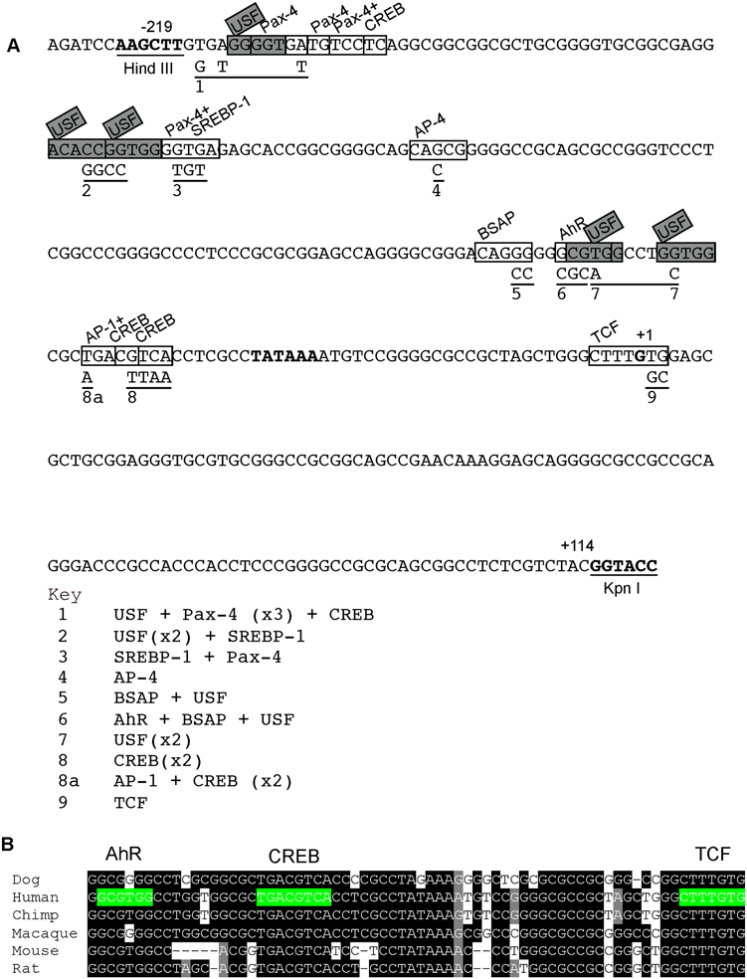
Identification of candidate transcription factor binding sites in the −219/+114 5′ flanking region of *FSCN1*. A, The DNA sequence of the −219/+114 region of *FSCN1* (from GenBank NC_000007.12). Candidate core motif binding sites for the indicated transcription factors were identified with the Match 1.0 Public, rVISTA or Target explorer algorithms, in combination with evaluation of the evolutionary conservation of the identified motifs in six mammals (see Figure S1 and Fig. 1). Motifs selected for mutagenesis are numbered 1–9 and are shown boxed. Nucleotide substitutions introduced in the point mutants are shown below the sequence. Nucleotides corresponding to the TATA box and predicted transcriptional start site are in bold. Restriction enzyme sites added for subcloning of the parent wildtype DNA are in bold and underlined. B, multiple sequence alignment demonstrating the conservation of the AhR, CREB and TCF motifs (motifs 6, 8 and 9) in mammals.

As demonstrated by the bioinformatics analyses, the region −219/+114 is well- conserved in mammals (Figure S1), and contains multiple conserved motifs (Fig. 1B, conserved region A). To identify candidate transcription factor binding motifs within this region of *FSCN1*, DNA sequences from the six mammals corresponding to −219/+114 of human *FSCN1* were analysed individually by Match 1.0 Public and pairwise by rVista (see Methods). Core candidate motifs identified with probability scores >0.8 in human and at least one other species were chosen for further experimental analysis. Overall, multiple candidate transcription factor binding sites were identified that met these criteria (Fig. 4A). Of these, only the CREB and TCF motifs were completely conserved across all six species and the AhR motif was highly conserved (Fig. 4B). These motifs also corresponded to conserved motifs identified by FootPrinter (Fig. 1). Additional candidate motifs identified in the human sequence included multiple USF or Pax-4 (paired box 4) sites, a SREBP-1 (sterol regulatory element-binding protein1) site and an AP-4 site (Fig. 4A). For clarity, we will refer to the motifs as motifs 1 to 9, with motif 1 being the most 5′ to the transcriptional start site (Fig. 4A). With the exception of the Pax-4 motif (motif 3), the additional motifs were conserved only in primates, or, in the case of motifs 1 and 5, between human and chimpanzee only.

**Figure 5:**
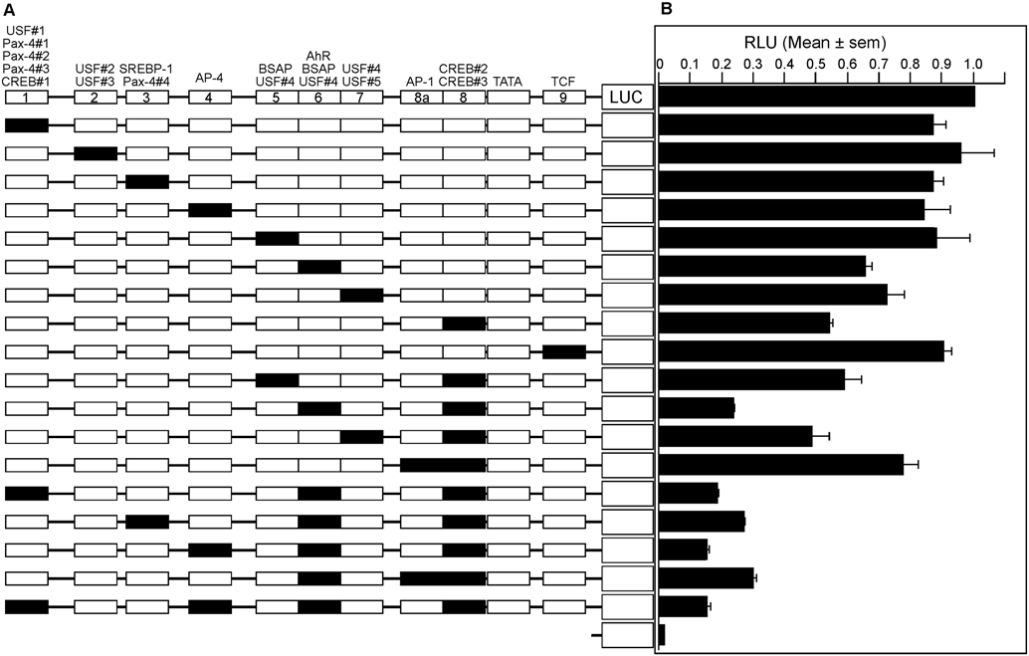
The transcriptional activity of *FSCN1* −219/+114 promoter region is due to multiple sequence motifs. A, Schematic view of the single and combined point mutations prepared in the *FSCN1* −219/+114 luciferase reporter construct. In the top line, each numbered box represents the correspondingly numbered motif, as shown in Fig. 4. The candidate transcription factor binding sites disrupted by each point mutation are listed above each box. Each lower line represents a different construct; black boxes represent the mutated motifs. Not to scale. B, Comparative analysis of the transcriptional activity of the point mutant *FSCN1* −219/+114 promoter reporter constructs. All constructs were analysed by luciferase reporter assay in SW480 cells, with normalization to Renilla luciferase activity. The mean normalized activity of wild-type *FSCN1* −219/+114 is set as 1 and the activities of the mutant constructs are expressed as a fraction of the wildtype. Each column represents the mean of 3 to 4 independent experiments, bars indicate s.e.m. Significant *p* values are stated in the text.

To test the functional relevance of the candidate transcription factor binding sites, point mutations were prepared to inactivate each of the identified conserved motifs in the context of the −219/+114 *FSCN1* luciferase reporter plasmid (Fig. 4A, Fig. 5A). The luciferase reporter activity of the mutants was compared to that of wild-type *FSCN1* −219/+114 in SW480 cells (Fig. 5B). Mutation of the candidate AhR binding motif (motif 6, Fig. 4A, Fig. 5A), resulted in a significant (*p* = 0.0305) reduction in transcriptional reporter activity. Similarly, mutations of the candidate USF motif (motif 7, *p* = 0.03), the CREB binding motif (motif 8, *p*<0.0001), or the TCF motif (motif 9, *p* = 0.004) each resulted in significantly reduced transcriptional reporter activity compared to wild-type (Fig. 5B). In contrast, mutation of sites 1, 2, 3, 4 or 5 had no statistically significant effect on luciferase reporter activity (Fig. 5B). The general significance of these results was assessed by repeating the analysis in COS-7 and MDA-MB-231 cells. Identical results were obtained, with the exception of the TCF motif (motif 9), for which the mutation did not reduce transcriptional reporter activity in COS-7 cells (data not shown).

Because several of the point mutants decreased but did not abolish transcriptional reporter activity in multiple cells, selected combinatorial point mutants were generated and tested in SW480 cells. Double mutations of the BSAP and CREB motifs; USF and CREB motifs, or the AP-1 and CREB motifs, did not further decrease reporter activity compared to the CREB motif mutant alone (Fig. 5B). In contrast, the combined mutation of the AhR and CREB motifs inhibited transcriptional activity very strongly in comparison to the individual mutations (*p* = <0.0001 vs the single AhR mutant and *p* = 0.0002 vs the single CREB mutant) (Fig. 5B). It was not possible to combine the designed AhR and USF motif mutations because the core motifs overlap extensively in the nucleotide sequence (Fig. 4A).

In view that the double mutant of the AhR and CREB motifs retained a low level of reporter activity, a number of triple point mutants were tested. Combining the motif 1 mutant had only a minor additional effect (Fig. 5B). Inclusion of either the SREB site (motif 3) or AP-1 site (motif 8a) mutant with the AhR and CREB motif mutants did not further decrease reporter activity (Fig. 5B). However, inclusion of the AP-4 site (motif 4) mutant with the AhR and CREB motif mutants resulted in a statistically significant reduction of activity (Fig. 5B, *p* = 0.02 vs the double AhR and CREB motif mutant). Reporter activity was not further decreased in a quadruple mutant that included the motif 1 mutation (Fig. 5B). These experiments were repeated in COS-7 cells with identical results (data not shown). These data identify the candidate AhR and CREB motifs as major determinants of the transcriptional activity of the *FSCN1* −219/−43 region.

### The transcription factors CREB and AhR are specifically associated with the −219/+7 region of *FSCN1* in fascin-positive human carcinoma cells

**Figure 6:**
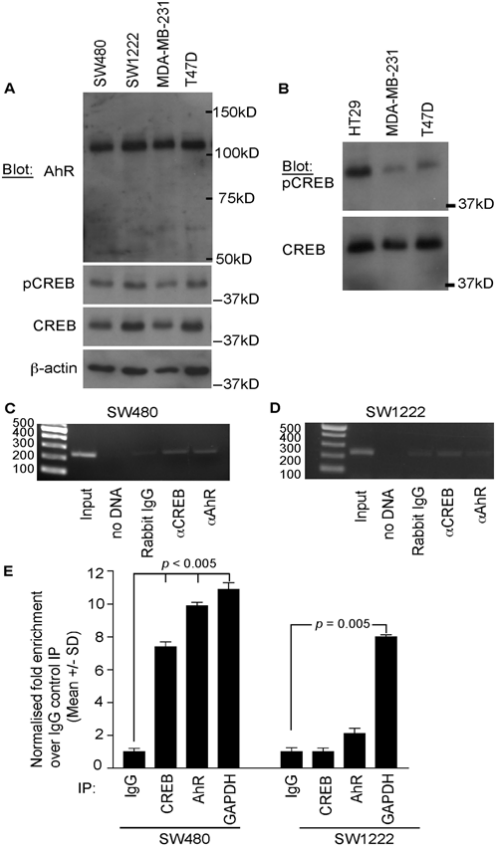
CREB and AhR are specifically associated with the −219/+7 region of *FSCN1* in fascin-positive human colon carcinoma cells. A, B, expression of AhR and CREB transcription factors in human carcinoma cells. Whole cell extracts from the equivalent of 4.5×10^4^ cells/lane were resolved on 10% SDS-PAGE gels under reducing conditions, transferred to PVDF membrane and immunoblotted with the indicated antibodies. C, D, detection of CREB and AhR binding to the −219/+7 region of the FSCN1 promoter by chromatin immunoprecipitation from SW480 cells [C] or SW1222 cells [D], as analysed on agarose gels. Sizes of markers are given in nucleotides. E, Bar graph from qRT-PCR analysis showing the relative fold enrichment of the −219/+7 FSCN1 promoter region in test immunoprecipitates relative to the control IgG immunoprecipitate, which is assigned a value of 1. Values are the mean+/−SD from duplicate reactions and 3 separate experiments.

On the basis of the above experimental data, and the very strong evolutionary conservation of the candidate CREB and AhR binding motifs between the six mammals (Fig. 4B), we next examined whether CREB and AhR specifically associate with the endogenous *FSCN1* promoter in human carcinoma cells. Immunoblot analyses with well-characterised antibodies demonstrated that AhR and CREB were both expressed in all four of the carcinoma cell lines (Fig. 6A). The molecular mass of AhR was consistent with that reported in other studies [33], [34]. Phosphorylation of CREB at residue Ser-133 promotes recruitment of coactivators needed for transcriptional activation [35], and there was no detectable difference in the content of S133-phosphoCREB in the four lines of fascin-positive or –negative human carcinoma cells (Fig. 6A). The antibody used is a sensitive detector of pCREB [36], [37] and indeed differences in pCREB content were apparent when additional cell lines were compared (shown for HT29 cells in Fig. 6B). By immunofluorescence, AhR, CREB and pS133-CREB were located principally in the nuclei of SW1222 and SW480 cells (data not shown).

We used chromatin immunoprecipitation to examine whether AhR and CREB are associated in SW480 cells with the endogenous 5′ flanking region −219/+7 of *FSCN1*. Histone binding to the promoter region of the glyceraldehyde 3-phosphate dehydrogenase *(GAPDH)* gene was used as a positive control (data not shown). AhR and CREB were each reproducibly detected in association with the −219/+7 region of the endogenous *FSCN1* promoter and were enriched relative to the negative control immunoprecipitations (Fig. 6C). To examine whether these associations were specific to fascin-positive carcinoma cells, the analysis was extended to SW1222 cells. In immunoprecipitates from chromatin extracts of SW1222 cells, no enrichment of AhR nor CREB in association with the −219/+7 region of the *FSCN1* promoter was detectable relative to the negative control imunoprecipitation (Fig. 6D). To confirm the specificity of immunoprecipitation, the PCR products from the input samples of both SW480 and SW1222 cells were sequenced: both DNAs had exactly the same sequence that corresponded to the human genome reference sequence for *FSCN1* as shown in Fig. 4A.

To quantify the association of CREB and AhR with the −219/+7 region of the *FSCN1* promoter in SW480 and SW1222 cells, quantitative real-time PCR (qRT-PCR) was carried out. DNA samples from chromatin immunoprecipitates from SW1222 and SW480 cells were analysed in comparison to the respective input DNA and DNA from negative control IgG immunoprecipitates, all in the same sets of reactions. For the reactions from SW480 cells, the CREB and AhR immunoprecipitations, and GAPDH used as a positive control, all resulted in significant enrichment of the −219/+7 promoter region relative to IgG control immunoprecipitations (Fig. 6E). For the reactions from SW1222 cells, only the GAPDH positive control was significantly enriched relative to negative control immunoprecipitations. A small enrichment for the AhR immunoprecipitations did not reach statistical significance (p = 0.38) (Fig. 6E).

These results demonstrate a correlation between the association of CREB and AhR with the −219/+7 region of the FSCN1 promoter and fascin protein expression in human colon carcinoma cells.

### Association of β-catenin with the −219/+114 region of human *fascin-1* is not specific to fascin-positive carcinoma cells

**Figure 7:**
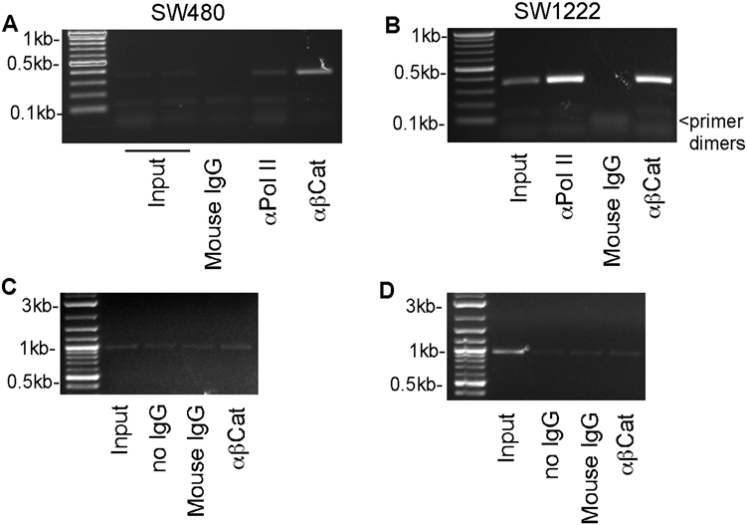
Analysis of β-catenin association with the *FSCN1* promoter region in fascin-positive and –negative human colon carcinoma cells. A, B, analysis of β-catenin binding to the −219/+114 region of the FSCN1 promoter by chromatin immunoprecipitation from SW480 cells [A] and SW1222 cells [B]. Immunoprecipitation with RNA polymerase II antibody was used as a positive control and is shown in each set. C, D, analysis of β-catenin binding to the −2384/−1412 region of the *FSCN1* promoter by chromatin immunoprecipitation from SW480 cells [C] and SW1222 cells [D]. No specific association was detected.

There are conflicting reports in the literature on the relevance of β-catenin signaling for regulating *fascin-1* promoter activity [3], [8]. In view that the candidate TCF binding motif (motif 9, Fig. 4) is highly conserved in mammals and that mutation of this site affected the transcriptional reporter activity of the −219/+114 region in SW480 cells, we examined the possible association of β-catenin with this region of the *FSCN-1* promoter and its specificity. By chromatin immunoprecipitation from SW480 extracts, the −219/+114 region was specifically enriched in β-catenin immunoprecipitates compared to negative control immunoprecipitates (Fig. 7A). However, the identical result was obtained with chromatin immunoprecipitates prepared from fascin-negative SW1222 cells (Fig. 7B). Furthermore, β-catenin was also found to be specifically associated with the −219/+114 region of the *FSCN1* promoter in fascin-negative Namalwa B lymphocyte cells (data not shown). Thus, the association of β-catenin with the −219/+114 region was not found to be specific to fascin-positive human carcinoma cells. From the bioinformatic analyses, we had identified a second candidate TCF binding motif that is conserved in human, chimpanzee and dog (Fig. 1, position indicated by asterisk). However, by chromatin immunoprecipitation, no specific association of β-catenin with this region of the genome was detected in either SW480 or SW1222 cells (Fig. 7C, 7D) relative to the negative control immunoprecipitations. Collectively, these data do not support the hypothesis that β-catenin has a specific role in regulating *FSCN1* transcriptional activity in fascin-positive human carcinoma cells.

## Discussion

Because fascin up-regulation correlates with poor prognosis and metastatic progression in multiple human carcinomas, fascin is emerging as an attractive potential therapeutic target. We combined bioinformatic and experimental approaches to identify motifs within the *FSCN1* promoter that are of functional relevance to the pathological expression of fascin in human carcinomas. By comparing the *fascin-1* promoter region from six mammalian species, we obtained resolution for identification of candidate regulatory motifs in the human *fascin-1* promoter. Similarly, distinctions between the human and mouse *fascin-1* promoters that have been obscure from analysis of either species could be clarified. The central novel findings of our study are: a), the identification of two separate regions within the 5′ flanking region of mammalian *fascin-1* genes in which specific, highly conserved sequence motifs are concentrated; b), the identification of a combined action of the well-conserved CREB- and AhR-binding motifs in positive transcriptional regulation of *FSCN1*, and c), the identification that AhR and CREB transcription factors are specifically associated with the *FSCN1* promoter in fascin-positive human colon carcinoma cells.

The availability of multiple sequenced mammalian genomes has greatly increased possibilities for making broadly based, and therefore more accurate, identification of evolutionarily conserved features within the human genome. This is particularly important for analyses of candidate transcription factor binding sites, for which the core motifs are under 10 nucleotides long, and which tend to be over-predicted by motif identification algorithms. With the benefit of genomic sequences from six mammals for multiple sequence alignment and motif analysis, we definitively identified two separate major regions of very high sequence conservation situated within 2 kb upstream of the translational start codon of *fascin-1*. Both regions contain multiple conserved motifs located in a conserved order, suggestive of important functional roles in transcriptional regulation of *fascin-1*.

Indeed, through analysis of the activity of a set of promoter reporter constructs we have demonstrated a central importance of the *FSCN1* promoter region −219/+114, (that contains conserved region A), in providing positive transcriptional regulation in human carcinoma cells. We identified a commonality of regulatory mechanisms between fascin-positive breast and colon carcinoma cells and human fibroblasts that are constitutively fascin-positive. This implies that fascin up-regulation in carcinomas is likely to be mediated by aberrations in normal regulatory mechanism(s), rather than activation of a novel mechanism. We note that the basal low level of promoter reporter activity in SW1222 and T47D cells is in contrast to the complete absence of fascin protein in these cells ([4] and Fig. 6A of this study). We considered that DNA methylation might contribute to transcriptional repression of the endogenous *fascin-1* gene in these cells. However, no increase in fascin protein was detected in SW1222 cells treated with the DNA methylation inhibitor 5-azacytidine for up to 72 h (unpublished observation). Other epigenetic mechanisms that would also be deficient in the plasmid context include other histone modifications or effects of chromatin conformation [38].

These results can be contrasted with a previous study of the activity of transcriptional regulatory elements in the *FSCN1* promoter in human dendritic and monocyte cells. In this study, *FSCN1* promoter reporter activity also correlated with the natural level of fascin protein in the cells; however, distinct transcriptional regulatory mechanisms were identified in the different cell types [26]. The *FSCN1* promoter region −1600/−210 was found to contain positive regulatory activity in mature dendritic cells, whereas in monocytes or neuronal cells this region included repressor activity [26]. The identification of repressor activity within the region distal to −210 is comparable with our study. No transcriptional repressor proteins have yet been identified for the *FSCN1* promoter, although it is of interest that activation of liver X receptor in lipopolysaccaride-matured dendritic cells reduces fascin protein levels [39]. However, a general conclusion emerging from the study with dendritic cells and our study is that, in all the cell types examined in both studies, the region −219/+114 has major transcriptional activity.

The presence of candidate CREB- and AhR-binding motifs in proximity to the *FSCN1* transcriptional start site has been noted [26], but the roles of these motifs or the relevant DNA-binding transcription factors in promoter activity were not investigated previously. Through unbiased methodologies for analysis of sequence conservation within the −219/+114 *FSCN1* promoter region, combined with experimental analysis of the effects of point mutations on promoter reporter activity, we establish for the first time that the CREB- and AhR-binding motifs each contribute substantially to transcriptional activity of the *FSCN1* promoter. The combined mutation of both motifs inhibited promoter reporter activity most strongly. We substantiated the concept of a direct functional role for the CREB and AhR transcription factors by the following novel findings: 1) both proteins are physically associated with the −219/+7 *FSCN1* promoter region in fascin-positive SW480 human colon carcinoma cells; 2), as confirmed by qRT-PCR, these associations are not detectable in fascin-negative SW1222 human colon carcinoma cells. It should be noted that the chromatin immunoprecipitation method does not identify the precise transcription factor binding site on the DNA; additional approaches such as EMSA or use of promoter decoys will be required to confirm this point. CREB binds as a dimer to DNA and then recruits either CBP or p300 as a co-activator of transcription [22]. CBP has been demonstrated to impact the levels of *fascin-1* transcript and protein in differentiating NT2 neuronal cells and is thus a plausible candidate for a co-activator of CREB on the *FSCN1* promoter in SW480 cells [27].

Increased expression of CREB is correlated with high grade prostate carcinomas [40], however, there are limited data on the relationship between CREB expression and the progression and/or metastasis of colon carcinomas. Mechanisms of CREB activation are complex and involve cAMP-activated phosphorylation of CREB plus additional processes to generate signaling specificity [32], [41]. Thus, we consider it unlikely that there is a simple relationship between levels of CREB, pCREB, and tumour status. Interestingly, both CBP and p300 are frequently mutated in colon cancer cell lines with microsatellite instability [42]. Microsatellite instability is most frequent in tumours of the proximal colon, which are the tumours that most frequently over-express fascin [14], [17]. We hypothesise that up-regulation of fascin transcription in colon carcinomas might take place in conjunction with altered regulation of the CREB complex. Further complexity is indicated by our finding that AhR binding to the −219/+7 *FSCN1* promoter region also regulates promoter activity. AhR is best known for its roles in pathways of metabolism of environmental chemicals, such as dioxin, to toxic or carcinogenic intermediates. Roles of AhR in the regulation of cell proliferation, apoptosis, and cell adhesion and migration are now emerging [43]. These activities have been proposed to contribute to the progression of mammary tumours [44]. Ligand-independent AhR up-regulation has been documented in tumours and linked with promotion of cell proliferation (e.g., [45]). Similar to CREB, AhR functions in the context of transcriptional coactivators or corepressors: it will be of future interest to identify the upstream signaling mechanisms by which CREB and AhR binding to the *FSCN1* promoter are activated in carcinoma cells.

We also analysed the possible link between β-catenin signaling and *FSCN1* promoter activity. This pathway has been proposed for a number of years but, to date, has only been analysed with reference to the mouse *fascin-1* promoter [3], [8]. Under conditions of active β-catenin signaling, β-catenin associates with DNA through its interaction with LEF1/TCF DNA binding transcription factors [28]. Although multiple candidate TCF binding sites can be identified in the individual mouse or human *fascin-1* promoter sequences ([3], [8]; our observations), phylogenomic analysis of conserved motifs demonstrates that, in fact, only one TCF site is conserved between primates and mice. This site is located at the proposed transcriptional start site (Fig. 1, motif 9 in Fig. 4A). Experimentally, we found that an inactivating point mutation of this site altered −219/+114 *FSCN1* promoter reporter activity in SW480 cells but did not affect promoter activity in COS-7 cells. Furthermore, chromatin immunoprecipitation for β-catenin carried out in both fascin-positive and negative- human colon carcinoma cells demonstrated that an association of β-catenin with the endogenous −219/+114 promoter region did *not* correlate with fascin protein levels. The specificity of this result was confirmed by examination of a more distal region containing a TCF binding motif conserved between human, chimpanzee and dog, for which no specific association of β-catenin was detected in either cell type. Overall, these data do not support the hypothesis that β-catenin signaling specifically regulates fascin expression in human carcinoma cells. The role of β-catenin in constitutive association with the *FSCN1* promoter is unclear at this time. However, β-catenin has been demonstrated to participate in transcription factor complexes that include additional components, such as CBP/p300, that exhibit both transcriptional repressor and activation properties [46]: we speculate that such an alternative complex may represent the context of β-catenin associated with the *FSCN1* promoter.

### Conclusion

We have identified that the conserved CREB and AhR binding motifs within the promoter region of the *fascin-1* gene are major and specific determinants of transcriptional activity, and that the association of these transcription factors with the corresponding *FSCN1* promoter region is specifically elevated in fascin-positive human colon carcinoma cells. These novel findings will guide further analysis of the environmental cues that activate aberrant expression of fascin in early stage human carcinomas.

## Materials and Methods

### Cell lines and other materials

SW480 and SW1222 human colon adenocarcinoma cells, human dermal fibroblasts (HDF) and COS-7 cells were cultured in DMEM containing 10% fetal calf serum (FCS). MDA-MB-231 and T47D human breast carcinoma cells were cultured in DMEM containing 5% FCS. All cells were maintained at 37°C in a humidified, temperature- and CO_2_-controlled incubator. Mouse monoclonal antibody to cAMP response element-binding protein (CREB) (86B10) and rabbit monoclonal to phosphoCREB-S133-PO_4_ (87G3) were from Cell Signaling. Rabbit polyclonal IgG to CREB used for chromatin immunoprecipitation was from Upstate. Rabbit polyclonal IgG to AhR was from Santa Cruz. Mouse monoclonal antibody to β-actin (AC-15) was from Sigma. Mouse monoclonal antibody to fascin (55k-2) was from Dako. Mouse monoclonal antibody to β-catenin (clone 14) was from BD Transduction Labs. Non-immune rabbit or mouse IgG was from Sigma.

### Bioinformatic analyses of the 5′ flanking regions of mammalian f*ascin-1* genes

Nucleotide sequences for 5.5 kb of DNA 5′ to the ATG codon of *fascin-1* genes from six mammalian species were obtained from the Entrez Genomes division of NCBI (http://www.ncbi.nlm.nih.gov/sites/entrez/dbgenomeprj). The sequences were from: *Homo sapiens* [47], Build 36.2, chromosome 7; *Pan troglodytes* [48] Build 2.1, chromosome 7; *Macaca mulatta* [49] Build 1.1, chromosome 3; *Canis lupus familiaris* [50] Build 2.1, chromosome 6; *Mus musculus* [51] Build 37.1, chromosome 5 (5 86.0 cM), and *Rattus norvegicus* [52] RGSC v3.4, chromosome 12.

DNA sequence conservation between the species was examined by multiple sequence alignment of a 2 kb region 5′ to the ATG codon, using TCOFFEE Regular [53] at EMBnet (http://ch.embnet.org/software/ClustalW-XXL.html). The sequences were also analysed by the algorithm FootPrinter 3.0 (http://genome.cs.mcgill.ca/cgi-bin/FootPrinter3.0/FootPrinterInput2.pl) that identifies short highly conserved regions, according to parsimony criteria in combination with the use of established phylogenetic relationships [29]. The motif size was set to 10 and a maximum parsimony score of 2 was used. Candidate transcription factor binding sites were identified in each sequence using Match 1.0 Public (http://www.gene-regulation.com/) with the algorithm set to mimimise false negatives [54]. Match 1.0 uses a library of mononucleotide position specific weight matrices from TRANSFAC® 6.0 to predict candidate binding sites for specific transcription factors [55]. Pairwise analyses were also made through rVISTA 2.0 (http://rvista.dcode.org/) in which pairwise sequence alignment is combined with analysis against the TRANSFAC library of matrices to identify conserved candidate transcription factor binding sites [56]. Because of the natural variety of TCF-binding nucleotide sequences, candidate TCF binding sites were also identified with the algorithm Target Explorer [57] (http://luna.bioc.columbia.edu/Target_Explorer/) using a customised position specific weight matrix based on a library of motifs, compiled from a previous analysis of TCF binding motifs [58] in combination with additional TCF motifs from the literature, with the minimum threshold set to 4. All results from the motif identification programmes were also examined against the TCOFFEE and FootPrinter sequence alignments.

### Construction of promoter reporter plasmids

Firefly luciferase cDNA, the gift of Donna Driscoll, CCF, was amplified by PCR with primers 294F/295R (Table S1). The PCR product was subcloned into pcDNA3.1/V5-His TOPO mammalian expression plasmid by the TOPO cloning method according to manufacturer's procedures (Invitrogen). This plasmid was designated pcDNA3.1Luc. The BAC clone CTB-161C1 (GenBank AC006483), that contains 196416 bp of DNA from human chromosome 7 including the region of *FSNC1* in pBeloBAC11, was from Invitrogen. BAC DNA was prepared with the QIAGEN Large DNA construct kit and used as a template for PCR to amplify portions of the 5′ flanking region of *FSCN1* using the primer pairs listed in supplementary Table 1. The entire 3.1 kb 5′ flanking region was sub-cloned into pcDNA3.1Luc by a multi-step cloning procedure. First, the region −2952/−946 was amplified with PCR primers 352F/369R, digested with BamHI and KpnI and ligated into BamH1/KpnI digested pBS-SK. The region −1512/+114 was amplified with PCR primers 357F/353R, digested with XcmI and KpnI and ligated 3′ to the −2952/−1512 region in pBS-SK digested with XcmI/KpnI. The 3.1 kb insert was digested from pBS-SK with BamHI and KpnI and subcloned into pcDNA3.1Luc from which the CMV promoter region had been removed by Bgl II/Kpn I digestion (Fig. 2; designated pcFSCN-2952/+114Luc). pcFSCN1-1582/+114Luc and pcFSCN1-219/+114Luc were generated by PCR with the primers listed in supplementary Table 1, followed by HindIII/KpnI digestion of the PCR product and ligation into correspondingly digested pcFSCN-2952/+114Luc. For the internal promoter deletion (Δ−1582/−50), PCR was carried out with primers 529F/530R, the product digested with AatII, and ligated in AatII-digested pcFSCN-2952/+114Luc. pcFSCN1-50/+114Luc was prepared by AatII digestion of pcFSCN-2952/+114Luc and self-ligation. pcFSCN1-43/+114Luc was prepared by AatII digestion of pcFSCN-2952/+114Luc, blunting of ends with T4 DNA polymerase, and self-ligation. All sequences were checked by automated DNA sequencing, carried out by CCF Genomics core facility.

### Preparation of point mutations in the *FSCN1* promoter

Sites for point mutation in the *FSCN1* 0.21 kb promoter region were selected based on the bioinformatic analyses of candidate transcription factor binding site core motifs and their conservation in six mammalian species. The mutations were designed based on known inactivating mutations of the motifs. All the designed mutant nucleotide sequences were re-analysed in Match 1.0 Public to check for loss of the specific transcription factor binding site and that no new binding sites were generated. The CREB binding site point mutant was based on previous inactivating mutations [59]. The TCF binding site point mutant was designed from the FOPFLASH plasmid sequence [60]. All mutations were prepared by PCR-based mutagenesis of the 0.21 kb promoter region in pcDNA3.1Luc plasmid, using the QuickChange II XL Site-directed mutagenesis kit (Stratagene) according to manufacturer's procedures and oligonucleotide primers as in supplementary Table 1. Sequences were confirmed by automated DNA sequencing of the entire 0.21 kb promoter region.

### Dual luciferase promoter reporter assay

1×10^5^ cells were plated per 14 mm well in 24-well plates for 24 hrs before transfection, with the exception of COS-7 cells that were plated for 6 hrs. All cells were transiently co-transfected with 5.5×10^−14^ moles of each *FSCN1* promoter-luciferase reporter construct and 0.034 µg of pRL-TK plasmid (Promega) using PolyFect (QIAGEN) according to the manufacturer's instructions. pRL-TK encodes *Renilla* (sea-pansy) luciferase and provided an internal control for transfection efficiencies. Appropriate amounts of pBlueScript plasmid were included in the co-transfection to maintain the total mass of DNA in each transfection at 0.34 µg. After 24 h, cell lysates were prepared and transcriptional activities measured using the Dual-luciferase reporter assay system according to manufacturer's instructions (Promega). Firefly and *Renilla* luciferase activities were measured in a MLX luminometer (DYNEX Technologies, Chantilly, VA). Relative firefly luciferase activity was calculated by dividing the absolute activity of firefly luciferase by the activity of *Renilla* luciferase. Within each cell line, the normalised reporter activity of the CMV promoter was set as 1, and the activities of the FSCN1 reporter constructs expressed as a fraction of the CMV activity. At least three independent experiments were carried out for all cells and constructs, with duplicate or triplicate samples in each experiment. Data were analysed statistically by unpaired Student's *t*-test.

### Chromatin Immunoprecipitation

The protocol for chromatin immunoprecipitation was based on the EZ-ChIP kit (Upstate) and manufacturer's procedures, with optimisation for the cell lines and antibodies tested. Briefly, SW480 or SW1222 cells were seeded in 10 cm dishes for 48 hours and protein-DNA complexes were cross-linked with 1% formaldehyde for 10 min. Cross-linking was quenched by addition of 125 mM glycine. Cells were washed with phosphate-buffered saline, harvested, resuspended in 1% SDS, 10 mM EDTA, 50 mM Tris.HCl, pH 8.1, lysis buffer containing protease inhibitors, and sonicated on ice by 6 pulses of 10 seconds duration each at the 30% amplitude setting of a BRANSON Digital sonifier (BRANSON, Danbury, CT). Soluble chromatin was collected by centrifugation and an aliquot taken to represent the input fraction. The remaining supernatant was incubated with 60 µl of protein G-Sepharose (50% v/v slurry) under rotation for 1 h at 4°C for pre-clearing. The supernatant was transferred to a new microcentrifuge tube, 4 µg of each antibody added with mixing for 5 h at 4°C, of for the β-catenin antibody 2 µg was used and the sample mixed overnight at 4°C. Protein G-Sepharose (40 µl of 50% v/v slurry) was then added for 1 h with mixing at 4°C. Bead pellets were washed for 5 min in 1 ml of low salt wash buffer, twice in high salt wash buffer, once in lithium chloride wash buffer, and twice in TE. Protein-DNA complexes were eluted in 200 µl of elution buffer and the cross-links reversed by overnight incubation at 65°C. DNA was purified using QIAamp DNA mini kit (QIAGEN) and eluted in 75 µl of 10 mM Tris.Cl, 0.5 mM EDTA, pH 9.0. 4 µl aliquots were used as templates for PCR amplification with primers 601F/603R (for CREB and AhR), or 353F/354R or 383F/384R (for β-catenin) and 449F/450R (for GAPDH). Reactions were carried out with annealing at 58°C and Hercules DNA polymerase (Stratagene) for 29 cycles. Reaction products were analysed on 1.5% agarose gels or by quantitative real-time PCR. Amplified DNAs were identified as the correct genomic regions by DNA sequencing.

### Quantitative real-time PCR

Reactions were performed on input DNA samples and DNA from chromatin immunoprecipitations prepared from SW1222 and SW480 cells using the Express SYBR GreenER qPCR SuperMix Universal (Invitrogen) and were run on an Prism 7000 instrument (Applied Biosystems) using 25 µl reaction volumes. Cycling conditions were 50°C for 2 mins, 95°C for 2 mins, followed by 40 cycles of 95°C for 15 sec and 60°C for 1 min. The relative proportion of immunoprecipitated promoter fragments was determined from the threshold cycle (C^T^) for each PCR reaction after normalization relative to input DNA. Fold enrichment in the test immunoprecipitations was calculated as two to the power [mean C^T^ value for that sample minus the mean C^T^ value from the IgG control immunoprecipitation] and were analysed statistically against the IgG control immunoprecipitation by two-tailed *t* test.

### Gel electrophoresis and immunoblotting

Whole cell lysates were prepared in SDS-PAGE sample buffer buffer (2% SDS, 10% glycerol, 50 mM Tris-HCl, pH 6.8). All samples were electrophoresed on 10% polyacrylamide gels under reducing conditions and transferred to polyvinylidene difluoride membranes (Millipore) using a semidry transfer blot system. Blocking and washing with Tris-buffered saline containing 1% Tween 20 (TBS-T), 2% skim milk powder and 0.5% bovine serum albumin (Sigma) was used for all antibodies except the pS133CREB antibody, for which TBS-T containing 5% bovine serum albumin was used. After incubation for 1 h with primary antibodies, or over-night with the phospho-specific antibodies, blots were developed with alkaline phosphatase-conjugated secondary antibodies and enhanced chemiluminescence (ECL) as described [61]. Quantitative analysis was performed using NIH Image J version 1.38.

## Supporting Information

Figure S1 TCOFFEE multiple sequence alignment of the 5′ flanking region of the fascin-1 gene from six mammalian species. 2 kb of sequence was extracted from each of the indicated genomes. Black shading indicates identical nucleotides, grey shading indicates conservation in less than 50% of the sequences. Sequences are numbered with reference to the transcriptional start site as +1; in the human sequence the ATG codon is at +122.
(0.27 MB RTF)

Table S1 List of oligonucleotides used for PCR-based sub-cloning, mutagenesis, or chromatin immunoprecipitation analysis.
(0.02 MB RTF)
