# Supplementary material for: Fascin-1 Promoter Activity Is Regulated by CREB and the Aryl Hydrocarbon Receptor in Human Carcinoma Cells
Source: PLoS One. 2009 Apr 2;4(4):e5130. doi: 10.1371/journal.pone.0005130 (PMC2661145; doi:10.1371/journal.pone.0005130)
Supplement: Figure S1 — TCOFFEE multiple sequence alignment of the 5′ flanking region of the fascin-1 gene from six mammalian species. 2 kb of sequence was extracted from each of the indicated genomes. Black shading indicates identical nucleotides, grey shading indicates conservation in less than 50% of the sequences. Sequences are numbered with reference to the transcriptional start site as +1; in the human sequence the ATG codon is at +122. (0.27 MB RTF) [file pone.0005130.s001.rtf]

Figure S1. TCOFFEE multiple sequence alignment of the 5' flanking region of the fascin-1 gene from six mammalian genomes. 2 kb of sequence is shown from each genome, except for the chimpanzee where the available sequence is limited by an unsequenced segment. Black shading indicates identical nucleotides, grey shading indicates conservation in less than 50 % of the sequences. Sequences are numbered with reference to the transcriptional start site as +1; in the human sequence the ATG codon is at +122.


Dog        1 -C-CC-GGCC-----------------------------------------CTGATCCCA
Human      1 GTACCCAATCTAG-AGCAGGACAAACACCTATCACCTGCACTGGCAATGGACAGAGGACA
Chimp      1 -G-CAA-----------------------------------------AGGACAGAGGACA
Macaque    1 -CAC-CTGCATTG------------------------------GCAATGGACAGAGGACA
Mouse      1 -CCCTCAGCCCGGGAGCGT-------------------------------------GCCT
Rat        1 TGCCT--TCCATC-AT----------------------------------------GATG

Dog       17 GTAG--GC--GGGTC--TCAG---CATGC--TCC-----TGCCAGAGCAAGCA---CCCC
Human     60 GTG---GC--AGCCCCATCACC-AGAGGCATTCA-----AGCCAAGGCATTTTCTATC--
Chimp     18 GTG---GC--GGCCCAATCACC-TCAGGCATTCA-----CGCCAAGGCATTTTCTATC--
Macaque   29 GTG---GC--AGCCCCATCACC-AGAGGCATTCA-----AGCCAAGGCGTTTTCTTTCTT
Mouse     23 TTACCCTCCGAGCTC--CCAGCTTTGGGTTTTTG-----AGGCAAGGTTTCATATAGC--
Rat       18 GT---GGG--GAGAC--TGAAT-TCAGCCCTTCGGCCTGAGCGTATGCCTTTACTCTCTG

Dog       58 AC-----AGCCTTCTG--------------------------------------------
Human    107 -------GTCTATTTA--------------------------------------------
Chimp     65 -------TTCTATCTA--------------------------------------------
Macaque   78 TCTTTCTTTCTTTCTT----------------------------------------TCTT
Mouse     74 -------------------------------------------------------TTAGG
Rat       70 ---AGCCTTGGCTCTAGCTCCCGGTTTTGGGTTTTTGAGGCAAGGTTTATATAGCTTAGG

Dog       69 ------------------------------------------------------------
Human    116 ------------------------------------------------------------
Chimp     74 ------------------------------------------------------------
Macaque   98 T------------CT---------------------------------------------
Mouse     79 CTGGCCTGGCATTCAAGGTCATCTCAGAGGCCTAGGATGACCTTGTGTTGTCGGTTCTCC
Rat      127 CTGGCCTGGCATTCAAGGTCATCTCGGAAGCCTAGGATGACCTTATCCTCCTGGTTCTTT

Dog       69 ------GAAA--------T--GCCT--ACAGCT-ATT-CATCCGCCT----CTT--CTCT
Human    116 ------TCTATCTA----TCTATCTATCTATCT-ATC-TATCTATCTATCTATC-TAGAT
Chimp     74 ------TTTA------------TTTATCTATCT-ATC-TATCTATGTATCTATT-TAGAT
Macaque  101 -TTCTTTCTTTCTT----TCTTTCTATCTATCT-ATC-TATCTATCTATCTATC-TATCT
Mouse    139 GGCCTTTACCTCTGGTGTGCTGGC-GTTTGCCTTACCACACCCAGGCATCCCCCATAGCT
Rat      187 TGCCTTTATCTCTGGTGTGCTGAT-GTCTGCATTACCACACCCAGGCATCCCCCGTAACT

Dog      103 GAG-CCT--TGG-A-----AGTTGGCCGG-------AGGT-TT--------GG--GGTC-
Human    163 GAGATCT--TGCTA-----TGTTGCCCAG---GTTGAACTCCTGGCCTCAAGC--GATC-
Chimp    113 GAGATCT--TGTTA-----TGTTGCCCAG---GTTAAACTCCTGGCCTCAAGC--GATC-
Macaque  153 AGGATCT--TGCTA-----TGTTGCCCAG---GCTGAACTCCTGGCCTTAAGC--AATC-
Mouse    198 TAAGCCTAAAGCTCT---CGGATGGCCAGCACGCCCATGCCCTGACGTGAAGTTCAGTGA
Rat      246 TGAGCCTAAAGTTTGGGATGGATGGCCCGCACATCCATGATCTGATGTGATGCTAAATGA

Dog      135 TTT-CTAATT-A--GT---TGCAAGGCTTCTGG--GG--ACTG-GAGTGGGT--------
Human    210 CTC-CTGCTTCA--GCC-TCCCAAAGTGT-TGG--GATTTCAG-GTGTGAACC-ACTGTA
Chimp    160 CTC-CTACTTCA--GCC-TCCCAAAGTGT-TGG--GATTTCAG-GTGTGAACC-ACTGTA
Macaque  200 CTT-CTGCTTCA--GCC-TCCCAAAGTGT-TCG--GTTTTCAG-GCTTGAGCC-ACTGTA
Mouse    255 CTAATCAATACAGAGCGTTCACGGAGCCC-TGACACATAGCAG-CATTGACTTAACCTTA
Rat      306 CTAATCAATGCCAGGCATTTATGGAGCCC-TGACACATAGCAGGCATTGACG------TA

Dog      175 CCT-----CAGGGTAG----ACAGTCAATGC---CTG-AG-CTCCACGTCC-----CCAG
Human    261 CCTGGCTGGAGTGCAGTGGCACTGTCATAGCTCACTGCAGCCTCCACCTCCTGGGCTCAA
Chimp    211 CCTGGCTGGAGTGCAGTGGCACTGTCGTAGCTCACTGCAGCCTCCACCTCCTGGGCTCAA
Macaque  251 CCTGGCTGGAGTGCAGCGGCACTATCATAGCTGACTGCAGCCTCCACCTCCTGGGCTCAA
Mouse    313 ACAGCCTGGTGTGTAATGAGGCCAACTTGTC-----------------------------
Rat      359 ACAGCCTGGTGTGTAATGAGGCAAACGTGTC-----------------------------

Dog      216 G-GACTCTC-----AGACTCTCCTGAGATGGGGGATTTGTAGCTCTT--CTA---CA--A
Human    321 GGAATCCTCTTTCCTCAGCCTCCTGAGTAGCTGGGACCACAGGCATGCACCATCACACCC
Chimp    271 GGAATCCTCTTTCCTCAGCCTCCCGAGTAGCTGGGACCACAGGCATGCACCATCACACCC
Macaque  311 GGGATCCTCCTTCCTCAGCCTCCTGAGTAGCTGGGACCACAGGCACGTGCCACCACACCC
Mouse    344 ------------------------------------------------------------
Rat      390 ------------------------------------------------------------


Dog      263 AACAGAAACT-GAGGCTCAGAGAGGTT------AACTTGCTTA----AG-----GTCGGT
Human    381 AGCTAAACCTTGATTT-TTTCCATAGT------ATATGGCTCA----GG-----GCAGAG
Chimp    331 AGCTAAACCTTGATTT-TTTCCATAGT------ATATGGCTCA----GG-----GCAGAG
Macaque  371 AGCTAAACCTTGATTT-TTTCCATGGT------GTATGGCTCA----GG-----GTGGAG
Mouse    344 ----------CGATTC-TGCCCACGATGCGATGCTCTGGGTTAAACAGGTTTGAACAGAG
Rat      390 ----------TGATTC-TGCCCAAGAGGC----TCCTGGGTTAAACAGGTTTGAACAGAG

Dog      307 CACACA-GCT-GG---TG--AGATCC-----TGGGTT--TCAAGCCTAATAGGTA-ACAT
Human    425 CAAAGAAGCACAA---AA--ACATGTTGGCTTGCGGT--TGAGGGCTGATGGGAG-GGGG
Chimp    375 CAAAGAAGCACAA---AA--ACATGTTGGCTTGCGGT--TGAGGGCTGATGGGAG-GGGG
Macaque  415 CGAAGAAGGACAA---AA--ATAGGTTGGCTTGCAGT--TGAGGGCTGATGGGAG-GGGT
Mouse    393 TACCGGATGGCAGAGAGGGCA-CCCTCAGTATCGGGTTTTGGCTGTTACTGCTTCCAGTC
Rat      435 TACCGGATTGCAG----GGCATCCCTCAGTATAGGGTTTTGGGTGTTAATGCCTCCAGTC

Dog      352 CAAAATGAG--AAACTCTTGATGTTCTGGCTCTTGAGGGATGGGT--TTT---GTG-GGA
Human    477 TT--CTGGCCAAAGCCCAAAATTAACAGCC--ACAAC-GTCAGTG--TCT---GTG-GGA
Chimp    427 TT--CTGGCCAAAGCCCAAAATTAACAGCC--ACAAC-GTCAGTG--TCT---GTG-GGA
Macaque  467 TT--CTGACCAAAGCCCGAAATTAACAGCA--ACAAC-ATCAGTG--TCT---GTG-GGA
Mouse    452 TT--CTGACCTGCTCCCTCAAG-GCTGGTC--TTAAA-CTCAAAGCAATCCTCCTGTCCC
Rat      491 TT--CTGACCAGGTCCCCCAAG-GCTGGTC--TTAAA-CTCAAAG--ACC---CTG-CCC

Dog      404 GGGAAGACTAT----GGCCACTGCCTG-CCTCAGGTTCCAGGCCCTGCCGGTAAACCCAT
Human    526 GGGGTTGCCAG----GGGCG-TGCGGGTTCTGGGGCTCAAGGCCCTGCCGGTAAACCCAT
Chimp    476 GGGGTTGCCAG----GGGCA-TGCGGGTTCTGGGGCTCAAGGCCCTGCCGGTAAACCCAT
Macaque  516 GGGGTTGCCAG----GGGCA-TGCGGGTTCTGGGGCTCAAGGCCCTGCCGGTAAACCCAT
Mouse    506 TGCTTCTCAAGTTCTGGAAT-TACAAGTG-TGACTCACTGTACCCAGG-AATATGTGTCT
Rat      539 TGTCTCTCAAGTTCTGGGAT-TACAAGTT-TGACTCCCTGTGCCCAGG-AGTATGTGTCT

Dog      459 TTGAGCCACGATGGTG---A----------------------------------------
Human    581 TTGAAGCAGGATGGCAAGAA----------------------------------------
Chimp    531 TTGAAGCAGGATGGCAAGAA----------------------------------------
Macaque  571 TTGAAGCAGGATGGCAAGAA----------------------------------------
Mouse    563 TTATTACAGAGGAAGGAGAGCTCAGAGAGTGAGAGTAACCTGTGCAAAGTCCCACAGCAG
Rat      596 TTACTGCAGAGTGGGAAGAGCTCAGAGAGCCCCTGTGATCTGTGCAAAGTCCCACAGCGG

Dog      476 ------------------------------------------------------------
Human    601 ------------------------------------------------------------
Chimp    551 ------------------------------------------------------------
Macaque  591 ------------------------------------------------------------
Mouse    623 GAGAGGATGGCTGGCTATCGGCCGCGAACTTGGTGACCTTTCCTGTGACCTCTAAGGGAG
Rat      656 GAGAGGACGGCTAGCTATCAACCTCCTTCTTGCTGACCTTTTCTGTGACCTCTAAGGGAG

Dog      476 ------------------------------------------------------------
Human    601 ------------------------------------------------------------
Chimp    551 ------------------------------------------------------------
Macaque  591 ------------------------------------------------------------
Mouse    683 GAACTAGATGTCCCAGCAAGGATTCTAGGGACTTGTCAGGACTGACCTTTGATGATGGGA
Rat      716 GAACTTGATTTCTCAGAAAGGATGCTAAAGGCTTGTCAGAGCTGACCTTTGATGATAGGG

Dog      476 -GGTGACACCATCTTCCACTGGA---AGA-TAAAGA-AATCCCTGGAGGGGTTTGCCTGG
Human    601 -GGTGACACCATCTTCCCCCGC-------GCACTGAAAGCCCCTGGCTGGGATTGCCTGG
Chimp    551 -GGTGACACCATCTTCCCCCGC-------TCACTGAAAGCCCCTGGCTGGGATTGCCTGG
Macaque  591 -GGTGATACCATCTTCCCCCCCACCCCCTTCATTGAAAGCCCCTGGCTAGGATTGCCTGA
Mouse    743 AAGCCATGCCATCTCCCCTTCC-------CCTTTGATGGTCGCTGGAGGGGTTTGCCTGG
Rat      776 AAGGCACGGCGTCTACCCTTCC-------TC---------------------TTGCCTGG

Dog      530 AGGAAGACA-AGGCCCAGAC-CAA--C-----------CAGCT------GGGGAGCTA-G
Human    653 G-GCAGACACAGGCTCGGAC-CAG--CCCCAGCAATCCCAGTT--TATCAGCGAGCCG-G
Chimp    603 A-GCAGACAGAGGCTCGGAC-CAG--CCCCAGCAATCCCAGTT--TATCAGTGAGCCG-G
Macaque  650 G-GCAGACACAGGCTCAGAC-CAG--CCCCAGCAATCCCAGTT--TATCAGCAAGCCGGG
Mouse    796 G-ACAGAGATGCGTTTGGACACAGATCACCGTGAAGACTGGTCAGTTTCAGT-ACCCT-G
Rat      808 G-ACAGGGATCTATTTGGACTCGGATCACCCTAAGGACTGGTCAACTTCAGT-ACCCT-G

Dog      568 TTCAGTGCTCT---TTGCCTCTG------GCCCCAAGACCTTGTGGGCAGCTTGTGGTCA
Human    706 CTGAGGGCCCGGAGTTATCTCAGTGCCC-GGCCTGAGACCTTGTGGGCAGCCTGTGGTCA
Chimp    656 CTGAGGGCCCGGAGTTATCTCAGTGCCC-GGCCTGAGACCTTGTGGGCAGCCTGTGGTCA
Macaque  704 CTGAGGGCCCGGAGTTAGGTCAATGCCC-GGCCTGAGACCTTGTGGGCAGCCTGTGGTCA
Mouse    853 CCCA--GCCTGTGGCTGGG-AAGGGCCAGGGGCCCAGATCTTGTGGGCAGCCTGGGGTAG
Rat      865 CCCA--GCCTGTGGCTGGG-AAGGGCCAGG-GCCCAGATCTTGTGGGCAGCCTGTGGTAG

Dog      619 TGCTGGCATTCCAGGGGCTTGCTTGGCATGTGGGGAATGTCCCAGCAAAAGTC-CTGGCC
Human    765 TGCTGGCATTCCAGGGGCCT-TTTGGCATGTGGGGAATGTC-CAGGAAAAGCC-TCAGCC
Chimp    715 TGCTGGCATTCCAGGGGCCT-TTTGGCATGTGGGGAATGTC-CAGGAAAAGCC-TCAGCC
Macaque  763 TGCCGGCATTCCAGGGGCCT-TTTGGCATGTGGGGAATGTC-CAGGAAAACTCTTCAGCC
Mouse    910 GGCCAGCATTCCAGGGGCCTGCCTGGCATGTGGGGAATGTCCCAGGAGAAGTC-TCAGTC
Rat      921 GGCCAGCATTC-AGGGGCCTGCCTGGCATGTGGGGAATGTCGTAGGAGAAGTC-TCAGCC

Dog      678 TT-----------AGGAAAAGGAAGTGTTCCTAGAAGGGGGA--------GGAGAAGGCA
Human    822 TTCGGTGAGGCGCAGAAAAGGGAAGTGTCCCTAGAGGGGGTG--------GGTGAGGGCG
Chimp    772 TTCGGTGAGGCTCAGAAAAGGGAAGTGTCCCTAGAGGGGGTG--------GGTGAGGGCG
Macaque  821 TTCAGTGAGGCTCAGAAAAGGGAAGTGTCCCTAGAGGGGGCGGGTGGGGGGGTGAGGGCG
Mouse    969 CTCC-AGAAACTC---AAAGGGAAGTGTTCTTACGTGGGGTGG-TGTGT-GGTG-GGAGG
Rat      979 TTCC-AGAAACTC---AAAGGGAAGTGTTCTCATATGGGGTG-----------------G

Dog      719 GGG-GGTGGGTGTCTGCAGGGAATGCCCCCTTTGAGGGAGGAGTGTAAACAGTTGGGGTC
Human    874 TGGGAGGTGGTGTCTGCAGGGAATGTCCCCTTTGGGGGAGGAGGATGGAGGGTTGGGATT
Chimp    824 TGGGAGGTGGTGTCTTTAGGGAATGTCCCCTTTTGGGGAGGAGGATGGAGGGTTGGGATT
Macaque  881 TGGGAGGTGGTGTCCGTAGGGAATGTCCCCTTTG-GGGAGCAGAACTGAGAGTTGGGATT
Mouse   1022 TGGGAGGTGGTGTTTGT-GGG---------------------------------------
Rat     1018 TGGGAGGTGGTGTTTGT-GG----------------------------------------

Dog      778 CTGAAG---G-AGG---GCTTCTACCTTCCA-AACGTT-TTCCAGTGTGCCCAGCTCAGC
Human    934 CTGAGGATGGGGGGGGGGGCTGTAGCCAGCACCATGTCCCTCCTGTGTGACCAGCTCAGA
Chimp    884 CTGAGGATGGG----GGAGCTGTAGCCAGCATCATGTCCCTCCTGTGTGACCAGCTCAGA
Macaque  940 CTGAGGATGGG----GGAGCTGTAGCCAGCACCATGTCCCTCCTGTGTGACCAACTCAGA
Mouse   1042 ------------------------------------------------------------
Rat     1037 ------------------------------------------------------------

Dog      829 GACCTATGAAGTAGGAACCTGAGGGTATAAAGTAAGGGGCGCCCTAGTCCTGGAG-CTGT
Human    994 GTCCCATGAAATTGGGGCTTGGGAG-----GGGAAGGGACA--CTGGCCTGGGAACCAGA
Chimp    940 GTGCCATGAAATTGGGGCTTGGGAG-----GGGAAGGGACA--CTGGCCTGGGAACCAGA
Macaque  996 GTCCCATGAAATTGGGGCTTGGGAG-----GGGAAGGGACA--CTGGCTTGGGAACCAGA
Mouse   1042 --------------------------------AATGTCCCT--CTGGTCTGGAACCCTGA
Rat     1037 --------------------------------GATGTCCCT--CTGGTGTGGAACCCTGA

Dog      888 GA-CCTGGACCCTGCCCTTCAGGCTGTCAGA------------------CCAGGGCTGG-
Human   1047 GA-CCTGGGC--TGG---TCTGGCTCACAGT----------------------TGCTGG-
Chimp    993 GA-CCTGGGC--TGG---TCTGGCTCACAGT----------------------CGCTGG-
Macaque 1049 GA-CCTGGGC--TGG---CCTGGCTCACAGT----------------------TGCTGG-
Mouse   1068 GGGGCTG--C--AGT---TAGGAGGTACATTGGGGCTGTGCCATTCTAGG--GAGCTGAC
Rat     1063 GGGGCTG--C--CGT---TAAGAGTTACACT------------------G--GGGCTGAC

Dog      928 --TCCCACCTCTGATTAGC-CCAGTAAAAGGG-TCTGCTAACCCTGCCCTGCCAGAGCGC
Human   1078 --ACC--TCTGTGATCCGTGTCAAAAAACGAGAACACCAATTCCTGTCCTGCCCA-----
Chimp   1024 --ACC--TCTGTGATCCGTGTCAAAAAACGAGAACACCAATTCCTGTCCTGCCCA-----
Macaque 1080 --ACC--TCTGTGATCTGGGTCAAAAAACAAGAACACCAATTCCTGTCCTGCCCA-----
Mouse   1119 CTGCT--TTTCTGATGT--CTTCATAAACAGAGACCACTATTCCTGCCTTACCTA-----
Rat     1096 CGGCT--TTTCTGATGT--CTTCATAAACAAAGACCGCTGTTCCTGCCTTACCTA-----

Dog      984 CTGGGGGCTC-AGTCCTTTAGCGACTGCCTTAGCTCAGGTCATGATCTGGGGTCCTGGGA
Human   1129 -------CTC-A------------CCAC-------CAGGTG-GGACCTGA-ACCCTGGCA
Chimp   1075 -------CTC-A------------CCAC-------CAGGTG-GGACCTGA-ACCCTGGCA
Macaque 1131 -------CTC-A------------CTGC-------CAGGTG-GGACCTGA-ACCCTGGCA
Mouse   1170 -------CCCTG------------CTTC-------CTGGTA--GACCAGC-ACCCCGGGG
Rat     1147 -------CCCTA------------CTTC-------CTGGTA--GACCAGT-ACCCCAGCG

Dog     1043 TGGGTCCACTGAGTGCGGAGGCTGTTTCTCCTCTCTGCTCCCCTCGCCCTGATTGTGCAT
Human   1160 TCG--CCAGC-ATTGG-GA--ATGT---------CGGC--------CACTGACTCAA---
Chimp   1106 TCG--CCAGC-ATTGG-GA--ATGT---------CAGC--------CACTGACTCAG---
Macaque 1162 TCG--CCAGC-ATTGG-GA--ATGT---------GGGC--------CACTGACTCAG---
Mouse   1201 -CT--TGAGC-ATCAC-CA--CAGT---------AGGG--------ACTTTTGTGGG---
Rat     1178 -CT--TGAGC-TTCAC-CA--CAGT---------GGGG--------TCTTTTGTTGG---

Dog     1103 GTTCCCTCTCTCTCAAATAAATAAAATAAATAAATAAAATCTTACAAAAACAACATAAAA
Human   1194 ----CCACTCTCCCGGAGACCT---------------------ATTTGGGCCACCCGAGG
Chimp   1140 ----CCACTCTCTCAGAGACCT---------------------ATTTGGGCCACCTGAGG
Macaque 1196 ----CCACTATCCCGGAGACCT---------------------ATTTGGGCCACCTGGGG
Mouse   1234 ----CCACTGAC---------------------------------TTGGGCCACAGTTAC
Rat     1211 ----TCACTGAC---------------------------------TTGGGCCACAATCAC

Dog     1163 ACCCTGCACTG-CCT-ACCCCA-CTGCCTGGAAGACCAGAACCCTGAGGCTTCTGC-ATC
Human   1229 C----G-GGTG-CCT-GGGCCA-CAC---GGAGG-----GGTCCTG--------GTGGTC
Chimp   1175 C----G-GGTG-CCT-GGGCCA-CAC---GGAGG-----GGTCCTG--------GCAGTC
Macaque 1231 T----G-GGTG-CCT-GGGCCA-CAG---GGAGG-----GGTCC-G--------GTGGCC
Mouse   1257 CA--C-AATTTATTTAGAGTCAGCAA---GG-------------TG--------GCTGTA
Rat     1234 CA--C-AATTTATTTAGAGTCAGCAC---GG-------------TG--------GCTGTA

Dog     1219 CCCAGAATGGGAACAGTAGTCACTCATCTCTGCCGACCTCCTAGAACTATTTTGGGCCAG
Human   1265 TTCAG---GGCAGCGGCTGTGG-----GGCTGAA-GCCTCAAGGAACCACATC---TCTG
Chimp   1211 TTCAG---GGCAGCGGCTGTGG-----GGCTGAA-GCCTCAAGGAACCACATC---TCTG
Macaque 1266 TTCAG---GGCAGTGGCTGTGG-----GGCTGAA-GCCTCAAGGAACCACATC---TCTG
Mouse   1290 CTCTA---TGC--TGACCCGGG-----AATAGAA-ATCTCG---------GTT---TCTG
Rat     1267 CTCTA---TGC--TGGCCCTGG-----GATACAA-ATCTCA---------GTT---TCTG

Dog     1279 CAAGGGCTGGGTACTTGGGCCACAGGGAGGTGTCTGGGTGGCCTTCAGGGAAATGTTTTG
Human   1313 CA---------TAGGAGGGCCA--GG-------CTGCAGGGCCT-CGGAGA---------
Chimp   1259 CA---------TAGGAGGGCCA--GG-------CTGCAGGGCCT-CGGAGA---------
Macaque 1314 CA---------CAGGAGGGCCA--GG-------CTGCAGGGCCT-CGGAGA---------
Mouse   1327 CT---------CAGGAGGCCCA--GG-------CTACAAGGCCT-TG-AAA---------
Rat     1304 CT---------CAGGAGGCCCA--GG-------GTACAAGGCCT-TTGAGA---------

Dog     1339 CAAGGCCTGA-----GTTGGGTGAGTTGGGGTCTGC--AGG---CCCCTCCCCCAGCTCT
Human   1345 CA--ACCT-A-----GTTGGGCGTGTTGGGGTCTGT--GGG---TCCCAGGTCCTGGCCT
Chimp   1291 CA--ACCT-A-----GTTGGGCGTGTTGGGGTCTGT--GGG---TCCCAGGTCCTGGCCT
Macaque 1346 CA--ACCT-A-----GTTGGGTGTGCTGGGGTCTGT--GGA---TCCCAGGTCCTGGCCT
Mouse   1358 AA--CCCTGA-----GGCGGGC-----GGGGCAGGT--AGG---GCTCTGCAT-GCGCCT
Rat     1336 AA--CGCTAAGGCAGGTAGGGCAGGTAGGGCTCTTTATGCGCCTTTCTAGAGCTTAACTT

Dog     1389 -TCTGGCCTGCCTGGAC---CCCTCTGGCCCGCAGGCCCCTTGGGGTGCGGGTGGGGGG-
Human   1392 CACCGGGTCCCCACCGC---GCTGTCAGCTCCCAGCCTCTTTCCCTC-------GTCTGC
Chimp   1338 TACCGGGTCCCCACCGC---GCTGTCAGCTCCCAGCCTCCTTCCCTC-------GTCTGC
Macaque 1393 CACCGGGTCCCCACCGC---GCTGTCAGCTCCCAGCCTGCTTCCCTC-------GTCTGC
Mouse   1400 CTCTAGAAATTAACCTC----CTTTCTAC-CCCAGACC-CTT----C-------TGGCGT
Rat     1394 AACCTCTTTTCTACCCCAGACCCCTCTGTTTCCGGCCGGTATC-TTC-------TGGTGT

Dog     1444 -TCTC-CCTCCTTCATCGC--TCTGCTTGAAGGCCACCTCCTCCAAGCAGCCTCCGGACC
Human   1442 CTCTGGGCTTCTGTAAGGCTATGTGCTCCAAGGCCACCTCCTCCAGGCAGCCCTCAGACC
Chimp   1388 CTCTGGGCTTCTGTAAGTCTATGTGCTCCAAGGCCACCTCCTCCAGGCAGCCCTCAGACC
Macaque 1443 CTCTCGGCTCCTGAAAGGCTCTGTGCTCCAAGGCCACCTCCTCCAGGCGGCCCTCGGACC
Mouse   1443 CTCTGG-CTTCTTCAAGGAC--CGGCTCTAAGGCTACTTCCT---GGTACACCCTGGACC
Rat     1446 CTCTGG-CCTCTTCAAGGAA--CAGTTCTAAGGCTGGT----------ACACCCTGGACC

Dog     1500 ---GCCTCCACCCAGCACTGATGTGTTCTGTGGTCTTCGCGGT-----------------
Human   1502 CCCACCTTCGCCCAGTACCGATCTGCACCGTGGTCTCTGAAGT-----------------
Chimp   1448 CCCACCTCCGCCCAGTACCGATCTGCACCGTGGTCTCTGCGGT-----------------
Macaque 1503 CCCACCTCCGCTCAGTACCGATCTGCACCGTGGTCTCTG-GCT-----------------
Mouse   1497 CTC-CCTCCATCCTGAAGGGATCATTATCTTAAACCTGGTCCTTCCCATCGACTGGGTTT
Rat     1493 TTC-CCTCCATCCCGGACAGACCATTACCTTAAACCTGGTCCTTCCCAACGACTGGGCTT

Dog     1540 -------------GTCCGTCACAACCTCAAA--CCCGACTCGCCCTTCTTCCCCCCTATA
Human   1545 -------------CTCCATCGTGACCTCAAA--CCTCGCTCGTCCTTG----CTCCTAGC
Chimp   1491 -------------CTCCATCGTGACCTCAAA--CCTGGCTCGTCCTTG----CTCCTAGC
Macaque 1545 -------------CTCCATCGTGACCTCAAA--CCTGGCTCGTCCTTG----CTCCTAGC
Mouse   1556 GGGGGTAGGGGTGTTTCAGGATGGCCTCAGAGAGCAGGAGAAA-----------------
Rat     1552 GGGGGTAGGGGTGCTCCAGAATGGCCTCAGAGAGCAGGAAAAA-----------------


Dog     1585 GGGGCTCAG-GATGAGAGG-TGCCCCGAG----GGG---TCCCGGG-------CTGCGGT
Human   1586 GAGGCTTGG-GGTC-GGGG-TGTCCG-AGGTGGGGGACATCCGGGG-----GGGTTAGGT
Chimp   1532 GAGGCTTGG-GGTC-GGGG-TGTCCG-AGGTGGGGGACATCCGGGG-----GGGTTAGGT
Macaque 1586 GAGGCTTGG-GGTCAGGGG-CGTCCG-AGGTGAGGGACATCCGGGG-----GGGTTAGGT
Mouse   1599 ----GTTGGAGATCAGCCAACTACT-G---CAGAGCACGACTGTGGAGGAACAGTTAAAT
Rat     1595 ----TTTGGGGATCAGCCAACTACT-G---CA--GAACGACCGTGGAGGAAGAGTTAAAT

Dog     1629 GG---GGC----CCTGG-CACCCGGGGGCGCGGGCGGC--GGCGCGAG------CTGAGG
Human   1637 GG-CTGGC----GCGGG-GAGCCGGGGTTGTGAGGGGT--GATGTCCT------CAGGCG
Chimp   1583 GG-CTGGC----GCGGA-GAGCCGGGGTTGTGAGGGGT--GATGTCCT------CAGGCG
Macaque 1638 GG-CTGGC----GCGGG-GAGCCGGGGTTGGGAGGGGT--TGTGTCCT------CAGGCG
Mouse   1651 GGACTAGCAAACACGAACGGGCCAGAGGCAGAAGGACTGCTATGAGTTCGAGTCCAGCCT
Rat     1645 GGACTAGCAAACACGAACAGGCCAGAGGCAGAAGGATTGCTATGAGTTCGAGTCCAGCTT

Dog     1673 GCGCCGCGGAGG-CCGGC----GGCGCGGA----A--GCGGGGGGACGCCGAGGGCG---
Human   1683 GCGGCGCTGCGG-GGTGC----GGCGAGGACACCG--GTGGGGTGAGAGCACCGGCG---
Chimp   1629 GCGGCGCTGCGG-GGTGC----GGCGAGGACACCG--GTGGGGTGAGAGCAACGACG---
Macaque 1684 GCGGCGCTGCGG-GGTGG----GGCGAGGACACCG--GTGGGGTGAGAGCCCCGGCG---
Mouse   1711 G---AGCCACACAGATACTTCAAGCCTAGTCTGAGTTACAGAGTGAAACCC-GGTCTTAA
Rat     1705 G---AGCCACACATATACTTCAAGCCTAGTCTGGGTTGTAGAGTGAGACCCCCGTCTTAA

Dog     1719 ------GAA----CCGCCACCCGCGCCCCTCGGT-GCCGGCGCCGCGAGCTTCCCG-CGC
Human   1733 ------GGG----CAGCAGCGGGGG--CCGCAGC-GCCGGGTCCCTCGG---CCCGGGGC
Chimp   1679 ------GGG----CAGCAGCGGGGG--CCGCAGC-GCCGGGTCCCTCGG---CCCGGGGC
Macaque 1734 ------GGG----CAGCAGCGGGGG--CCGCAGC-GCCCTGTCCCTCGG---CCGGGCGC
Mouse   1767 ACCCCAGAAAAACACTCCG-GGGAA--GCGCAGGGGACCGGGCGCGCGC---TGCAGAGC
Rat     1762 ACCCCGGAAAAACCCGCAG-GGGAA--TCGCAGGGGCCCGGGACCACGC---TTCAGAGC

Dog     1767 CCCCTCCCCCGCCGAGCCCCGGGGCGGGGCGGGGCGGGGGCGGGGCCTCGCGGCGCTGAC
Human   1777 C-CCTCCCGCGCGGAGCC-AGGGGCGGGACAG---GGGGGCGTGGCCTGGTGGCGCTGAC
Chimp   1723 C-CCTCCCGCGCGGAGCC-AGGGGCGGGACAG----GGGGCGTGGCCTGGTGGCGCTGAC
Macaque 1778 CCCCACCCGCGCGGAGCC-GGGGGCGGGGAA-----GGGGCGGGGCCTGGCGGCGCTGAC
Mouse   1821 CCCCTCCCC-G-GCAGGCC-----CG------GGTAGGGGCGTGGCC-----ACGGTGAC
Rat     1816 CCCCTCCCCTG-GCAGTCC-----CG------GGGAGGGGCGTGGCCTAGC-ACGGTGAC

Dog     1827 GTCACCCCGCCTAGAAAGGGGCTCGCGCGCCGCGGG-CCGGCTTTGTGGAGCGCTGCGGA
Human   1832 GTCACCTCGCCTATAAAATGTCCGGGGCGCCGCTAGCTGGGCTTTGTGGAGCGCTGCGGA
Chimp   1777 GTCACCTCGCCTATAAAGTGTCCGGGGCGCCGCTAGCTGGGCTTTGTGGAGCGCTGCGGA
Macaque 1832 GTCACCTCGCCTATAAAGCGGCCCGGGCGCCGCGGGCCCGGCTTTGTGGAGCGCTGCGGA
Mouse   1863 GTCATCC-TCCTATAAAAC--CCTGGGCGCCGCCGGGCTGGCTTTGTGGAGAACTGCAGC
Rat     1863 GTCACCT-GCCTATAAAAC--CCATGGCGCCGCCGGGCTGGCTTTGTGGAGAGCTGCAGC

Dog     1886 GGGTGCGCGCCGGGCTGCGGTC-GCGAACAAAGGAGCCG-GGCGCCGCCGCGGGGACCCG
Human   1892 GGGTGCGTGC-GGGCCGCGGCAGCCGAACAAAGGAGCAGGGGCGCCGCCGCAGGGACCCG
Chimp   1837 GGGTGCGTGG-AGGCCGCGGCCGCCGAACAAAGGAGCAGGGGCGCCGCCGCGGGGACCCG
Macaque 1892 GGGTGCGCGC-GGGCCGCGGCTGCCGAACAAAGGAGCAGGGTCGCTGCCGCGGGGACCCG
Mouse   1920 GGGC------TAAGCCGT----GTTGAACAAAGGAGGTCG-------------GGCACAG
Rat     1920 GGAC------TAAGCGGT----GCTTAACAAAGGAGGTCC-------------GGAACAG

Dog     1944 CCACCG-ACCTCCCGG-GCCGCGCCGGGGCCCTCCCGCCCGCCGCCCGCCGCCACCATG
Human   1951 CCACCC-ACCTCCCGGGGCCGCGCAGCGGC-CTC-------TCGTCTACTGCCACCATG
Chimp   1896 CCACCC-ACCTCCCGGGGCCGCGCAGCGGC-CTC-------TCGTCTACTGCCACCATG
Macaque 1951 CCACCC-AACTCCCGGGGCCGCGCCGCGGC-CTC-------CCGTCCACCGCCACCATG
Mouse   1957 CTATCCAAGCTCCCGGGGCCAC--CG-GGC-----C-------GCCCTCCGCCACCATG
Rat     1957 CCACCCAAGCTCCCCGGGCTAC--CG-GGC-----C-------GCCCTCCGCCACCATG
