# Supplementary material for: Fascin-1 Promoter Activity Is Regulated by CREB and the Aryl Hydrocarbon Receptor in Human Carcinoma Cells
Source: PLoS One. 2009 Apr 2;4(4):e5130. doi: 10.1371/journal.pone.0005130 (PMC2661145; doi:10.1371/journal.pone.0005130)
Supplement: Supplementary file 2 [file pone.0005130.s002.rtf]

Supplementary Table 1. Oligonucleotide primers used in this study. All oligonucleotides were synthesised by Sigma-Genosys; oligonucleotides for mutagenesis were HPLC-purified.

Oligonucleotide	details	Sequence, 5' to 3'
294F	forward primer for luciferase cDNA	ACTGTTGGTAAAATGGAA
295R	reverse primer for luciferase cDNA (with Hpa I site)	GTACGTTAACCAATTTGGACTTTCCGCC
352F	forward primer for FSCN1 -2952/+114 (with Bam HI site)	CTCTGGATCCAAGCTTCTAGAAGGGGTATA
353R	reverse primer for FSCN1 -2952/+114 (with KpnI site)	ATATGGTACCGTAGACGAGAGGCCGCTGCG
357F	alternate forward primer for FSCN1 -2952/+114	ATGCACCATCACACCCAGCTAAAC
369R	reverse primer for FSCN1 -2952/+114 (with KpnI site)	TCAGGTACCCAACCCTCCATCCTC
529F	forward primer for FSCN1 -2952(D)/+114 (with AatII site)	GAAAAGTGCCACCTGACGTCGACG
530F	reverse primer for FSCN1 -2952(D)/+114 (with AatII site)	TGGAGACGTCAGTGAGCTATGACAGTGCCA
414F	forward primer for FSCN1 -1582/+114 (with HindIII site)	GTCAAAGCTTACTGCAGCCTCCACCTCCTG
413F	forward primer for FSCN1 -219/+114 (with HindIII site)	AGCCAAGCTTGTGAGGGGTGATGTCCTCA
589F	forward primer for mutagenesis USF, Pax-4 (motif 1)	GATCCAAGCTTGGGTGGGGTGTTGTCCTCAGGCG
590R	reverse primer for mutagenesis USF, Pax-4 (motif 1)	CGCCTGAGGACAACACCCCACCCAAGCTTGGATC
552F	forward primer for mutagenesis USF (motif 2)	GTGCGGCGAGGACAGGCCTGGGGTGAGAGCA
553R	reverse primer for mutagenesis USF (motif 2)	TGCTCTCACCCCAGGCCTGTCCTCGCCGCAC
535F	forward primer for mutagenesis SREBP-1 (motif 3)	GAGGACACCGGTGGGTGTAGAGCACCGGCGG
536R	reverse primer for mutagenesis SREBP-1 (motif 3)	CCGCCGGTGCTCTACACCCACCGGTGTCCTC
591F	forward primer for mutagenesis AP-4 (motif 4)	GGCGGGGCAGCACCGGGGGCCGCAG
592R	reverse primer for mutagenesis AP-4 (motif 4)	CTGCGGCCCCCGGTGCTGCCCCGCC
595F	forward primer for mutagenesis BSAP+USF (motif 5)	GGCGGGACAGCCGGGCGTGGCCTGG
596R	reverse primer for mutagenesis BSAP+USF (motif 5)	CCAGGCCACGCCCGGCTGTCCCGCC
554F	forward primer for mutagenesis AhR (motif 6)	CGGGACAGGGGGCGCTGGCCTGGTGG
555R	reverse primer for mutagenesis AhR (motif 6)	CCACCAGGCCAGCGCCCCCTGTCCCG
593F	forward primer for mutagenesis USF x 2(motif 7)	GGGGGGCGAGGCCTGCTGGCGCTGAC
594R	reverse primer for mutagenesis USF x2 (motif 7)	GTCAGCGCCAGCAGGCCTCGCCCCCC
481F	forward primer for mutagenesis CREB (motif 8)	CGCTGACTTAACCTCGCCTATAAAATGTCCG
482R	reverse primer for mutagenesis CREB (motif 8)	CGGACATTTTATAGGCGAGGTTAAGTCAGCG
582F	forward primer for mutagenesis CREB+AP-1 (motif 8a)	GCCTGGTGGCGCAGACTTAACCTCGCC
583R	reverse primer for mutagenesis CREB+AP-1 (motif 8a)	GGCGAGGTTAAGTCTGCGCCACCAGGC
568F	forward primer for mutagenesis TCF (motif 9)	TAGCTGGGCTTTGGCGAGCGCTGCGGAG
569R	reverse primer for mutagenesis TCF (motif 9)	CTCCGCAGCGCTCGCCAAAGCCCAGCTA
601F	forward primer for ChIP of FSCN1 (-223/+7)	GGGGTTGTGAGGGGTGATGTC
603R	reverse primer for ChIP of FSCN1 (-223/+7)	GCTCCACAAAGCCCAGCTAGC
413F	forwardprimer for β-χατενιν ΧηΙΠ οφ ΦΣΧΝ1 (-219/+114)	ΑΓΧΧΑΑΓΧΤΤΓΤΓΑΓΓΓΓΤΓΑΤΓΤΧΧΤΧΑ
353Ρ	ρεϖερσε πριμερ φβρχατενιν ΧηΙΠ οφ ΦΣΧΝ1 (-219/+114)	ΑΤΑΤΓΓΤΑΧΧΓΤΑΓΑΧΓΑΓΑΓΓΧΧΓΧΤΓΧΓ
383Φ	φορωαρδ πριμερ φορ ΧηΙΠ οφ ΦΣΧΝ1 (-2384/-1412)	ΑΧΑΓΑΓΓΤΓΧΧΧΑΤΧΓΤΑ
384Ρ	ρεϖερσε πριμερ φορ ΧηΙΠ οφ ΦΣΧΝ1 (-2384/-1412)	ΧΑΓΧΧΧΤΧΑΑΧΧΓΧΑΑΓΧ
449Φ	φορωαρδ πριμερ φορ ΧηΙΠ οφ ΓΑΠΔΗ	ΤΑΧΤΑΓΧΓΓΤΤΤΤΑΧΓΓΓΧΓ
450Ρ	ρεϖερσε πριμερ φορ ΧηΙΠ οφ ΓΑΠΔΗ	ΤΧΓΑΑΧΑΓΓΑΓΓΑΓΧΑΓΑΓΑΓΧΓΑ
